# Late Postoperative Evaluation of Retinal and Choroidal Thickness and Retinal Vessel Caliber after Surgical Repair of Corneal Perforation

**DOI:** 10.4274/tjo.57338

**Published:** 2015-12-05

**Authors:** Gökhan Pekel, Semra Acer, Nihal Cesur, Ramazan Yağcı, Ebru Nevin Çetin

**Affiliations:** 1 Pamukkale University Faculty of Medicine, Department of Ophthalmology, Denizli, Turkey

**Keywords:** Corneal perforation, macular thickness, peripapillary retinal nerve fiber layer thickness, choroidal thickness, retinal vascular caliber

## Abstract

**Objectives:**

To examine the late period retinal and choroidal alterations in the posterior pole of eyes that underwent primary suturing due to traumatic corneal perforation.

**Materials and Methods:**

This cross-sectional case series included 21 eyes of 21 patients. The fellow eyes served as the control group. Macular thickness, peripapillary retinal nerve fiber layer (RNFL) thickness, choroidal thickness, and retinal vessel caliber measurements were performed by spectral-domain optical coherence tomography (SD-OCT).

**Results:**

The mean RNFL thickness was 102.1±10.9 µm in the perforated eyes and 99.5±8.5 µm in the fellow eyes (p=0.29). The mean central macular thickness was 300.1±25.6 µm in the perforated eyes and 295.6±23.2 µm in the fellow eyes (p=0.62). The choroidal thickness and retinal vascular caliber measurements were also similar between the groups (p>0.05).

**Conclusion:**

Operated traumatic corneal perforations do not cause significant posterior pole retinal and choroidal SD-OCT thickness changes in the late postoperative period.

## INTRODUCTION

Corneal perforation is an important cause of ocular morbidity and vision loss, especially in developing countries.^1^ Various types of keratitis, trauma and some immune diseases are the main causes of corneal perforation.^2^ It is a surgical emergency that requires early repair of the wound to prevent possible blindness. There are several methods for the treatment of corneal perforation, including primary suturing, application of a bandage contact lens, gluing, amniotic membrane transplantation, the use of conjunctival flaps, and keratoplasty.^3,4,5,6^

If not properly managed, corneal perforation can lead to complications such as synechiae, glaucoma, cataract and endophthalmitis.^1,4^ Even after successfully operated corneal perforations, the visual quality and acuity of the patients do not reach the level of the fellow eyes. The reasons for low visual acuity are mainly irregular astigmatism and corneal opacities. Since the main problem is in the cornea, detailed macular and optic nerve examinations are sometimes underestimated.

In this study, we wanted to examine the posterior pole of patients who underwent operations due to corneal perforation. We performed our detailed posterior pole retinal and choroidal examinations after the removal of the corneal sutures in order to lessen astigmatism and increase measurement quality. Our aim was to reveal whether chronic macular edema, posterior pole atrophy, choroidal alterations, or any other retinal diseases occurred in the late stage of operated corneal perforation. We wanted to identify the late period retinal problems that might contribute to the low visual quality of these eyes.

## MATERIALS AND METHODS

Twenty-six patients who underwent surgical repair of corneal perforation not extending to the sclera were initially included. From these, five patients with low-quality optical coherence tomography (OCT) measurements were excluded. In total, 21 eyes of 21 patients who underwent surgery due to traumatic corneal perforations 3 mm or larger in size were evaluated. The fellow eyes of these patients were accepted as controls. The design of the study was a cross-sectional case series. The examinations were performed in a single tertiary referral center between 2012 and 2014. The study was conducted in accordance with the ethical standards of the Declaration of Helsinki and was approved by the Institutional Ethics Committee.

### Study Population

All of the patients underwent surgery to repair unilateral traumatic corneal perforations. The fellow eyes of the patients were healthy and served as the control group. The patients had open globe injuries only in Zone 1 according to the open globe injury classification system (i.e. cornea and limbus).^7^ Raw points in the ocular trauma score ranged from 66 to 76 for all the traumatized eyes.^7^ The patients who had corneal scars that impeded adequate retinal visualization were excluded. Scleral perforations were excluded, since the choroid, retina and vitreous were usually injured during the scleral perforation. Patients who had persistent high intraocular pressure (IOP) and/or endophthalmitis after surgery were also excluded. The patients had no systemic diseases and no history of ocular surgery in either eye before the corneal perforation repair.

### Surgical Techniques

All the operations were performed under general anesthesia within the first 24 hours after the ocular injury. Perforations were managed successfully with primary closure after maintaining the anterior chamber with viscoelastic substance. Glues, conjunctival flaps, amniotic membrane transplantation and corneal transplantation were not used in any of the patients. Corneal suturing was performed with 10/0 nylon sutures. In cases of anterior lens capsule rupture and cataract formation, lens removal was done by phacoemulsification in the first two weeks after the trauma. The corneal sutures were removed 3 to 4 months after the corneal wound repair.

### Measurement Techniques

**Figure 1:**
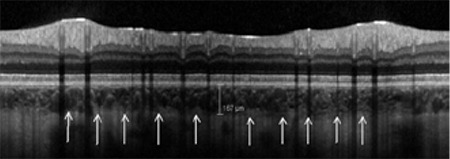
The peripapillary choroidal thickness measurement of one of the patients (the white arrows indicate the outer border of the choroid)

**Figure 2:**
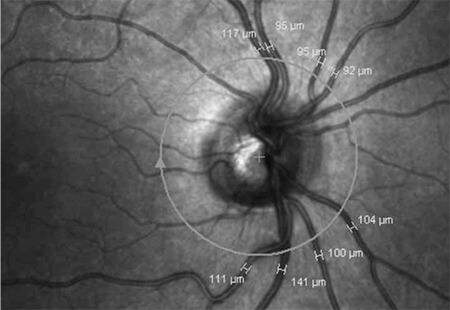
The retinal vascular caliber measurements of one of the patients

The examinations were performed in the late postoperative period (between 6 and 18 months after the operations). The rationale for this was to wait until corneal suture removal and corneal remodeling, since our aim was to find the reasons for low visual quality other than anterior segment problems (i.e., chronic macular edema, retinal nerve fiber layer (RNFL) atrophy). Spectral-domain OCT (Spectralis, Heidelberg, Germany) was used to analyze macular thickness and peripapillary RNFL thickness. In order to measure peripapillary choroidal thickness (PCT), a 360-degree 3.4 mm diameter peripapillary circle scan was performed using the standard protocol for RNFL evaluation, and only nasal PCT values were recorded for analysis. PCT was measured manually from the outer part of the hyperreflective line corresponding to the RPE to the inner part of the sclera (Figure 1). For retinal vascular caliber (RVC) analysis, retinal arterioles and venules passing through an area one-half to one-disc diameter from the optic disc margin were measured, and vessel calibers of the largest eight were selected for the analysis (Figure 2).

### Statistical Analysis

For statistical analysis, SPSS 17.0 software for Windows (SPSS Inc., Chicago, IL, USA) was used to analyze outcomes. Level of significance was accepted as α=0.05. The Mann-Whitney U test was used for comparison of studied parameters for the corneal perforated and fellow eyes. The Bonferroni correction was made when the patients with lens extraction were taken as a separate group.

## RESULTS

The mean age of the patients was 30.7±21.2 years (range, 6-75 years). The mean time interval between the corneal perforation operations and the posterior pole examinations was 11.1±5.0 months (range, 6-18 months). The etiologic agents of the corneal perforations were metallic objects in 10 eyes, wooden objects in 5 eyes, sharp edges of stones in 3 eyes, plastic objects in 2 eyes and glass particles in 1 eye.

Some of the demographic and clinical characteristics of the patients are shown in Table 1. The distribution of wound lengths was as follows: 3 mm in 3 patients, 4 mm in 9 patients, 5 mm in 5 patients, 6 mm in 2 patients and 7 mm in 2 patients. Six patients had anterior lens capsule rupture at presentation and underwent phacoemulsification within 2 weeks of the trauma. At the final visit, the mean best corrected visual acuity (BCVA) was 0.28±0.22 logMAR in the perforated eyes and 0.005±0.02 logMAR in the fellow (control) eyes (p<0.001). All the operated eyes had irregular astigmatism ranging from 1.50 to 8.00 diopters.

Segmental peripapillary RNFL thickness (inferior, superior, nasal and temporal) measurements are shown in Table 2. There was no statistically significant interocular difference in any of the RNFL quadrants. The mean RNFL thickness was 102.1±10.9 µm in the perforated eyes and 99.5±8.5 µm in the fellow eyes (p=0.29). The mean IOP was 14.0±2.7 mmHg in the perforated eyes and 13.7±1.7 mmHg in the fellow eyes (p=0.92). The mean central macular thickness was 300.1±25.6 µm in the perforated eyes and 295.6±23.2 µm in the fellow eyes (p=0.62). None of the patients had cystoid macular edema, RNFL atrophy, or any other retinal abnormalities.

The PCT and RVC measurements are shown in Table 3. The choroidal thickness and retinal arteriolar and venular caliber values were similar in the perforated eyes and the fellow eyes. We repeated all the analyses after excluding the patients with lens perforation, and again there were no statistically significant differences between the perforated and fellow eyes in the studied parameters (RNFL, macular thickness, PCT, RVC) (p>0.05). Also, there were no statistically significant differences when the patients who underwent traumatic cataract surgery were taken as a separate group (p>0.05).

## DISCUSSION

Our results show that operated clear corneal and limbal perforations do not cause chronic macular edema, peripapillary RNFL atrophy, choroidal thickness changes, or RVC alterations in the late post-operative period. Of course, this statement is valid only for uncomplicated corneal perforations that do not include posterior segment damage initially. To the best of our knowledge, this is the first report in the literature related to the impact of corneal perforation on posterior pole structures.

Although corneal perforation primarily affects the anterior segment of the eye, it is possible that retinal damage occurs by several mechanisms. In perforating corneal injuries, concomitant choroidal detachment was frequently seen, and this would have an impact on retinal morphology and function.^4^ Also, hypotony is common in corneal perforations both before and after surgical repair, so it is possible that hypotony maculopathy and its sequelae might occur to some extent. Since OCT is accepted as the best method for evaluating the posterior pole, we performed OCT in both eyes of the patients.

Our rationale for investigating RNFL was that optic nerve axonal loss is possible after severe ocular traumas, and this damage is shown clearly with spectral-domain OCT.^8^ The other mechanisms of peripapillary RNFL damage due to corneal perforation might be mechanical distortion of the eyeball during the injury causing optic nerve head damage, IOP fluctuations after the trauma, release of inflammatory mediators due to trauma and the effects of possible posterior pole edema. These factors might cause peripapillary RNFL atrophy in the chronic period.

Potential applications of OCT in posterior segment trauma have been reported in the literature,^9^ but there is lack of research about the indirect effects of anterior segment penetrating injuries on the retina using OCT. In this study, we did not observe any retinal abnormalities like cystoid macular edema, retinoschisis, retinal tears or macular holes in the late postoperative period. The macular thickness values of the perforated eyes and the fellow eyes were similar. Also, the peripapillary RNFL thickness of the perforated eyes did not differ much from that of the fellow eyes.

Singh et al.^4^ reported that choroidal detachment was present in nearly half of eyes with perforated corneal ulcers, occurring more frequently in larger perforations, and that it resolved after hypotony ended or by surgical drainage. It should be expected that some choroidal alterations as well as retinal vascular changes might occur in the late postoperative period of corneal perforation surgery. However, we did not find significant differences between the perforated eyes and the fellow eyes with respect to choroidal thickness or RVC.

The selection of an appropriate wound repair technique is mostly based on the size and location of the corneal perforation. If the corneal wound caused by a perforating injury is more than 3 mm in length, suturing is required.^2^ Gluing, bandage contact lens or amniotic membrane transplantation can be used for corneal perforations of 3 mm or less.^10,11,12^ Since the length of the wound was ≥3 mm in all the cases and concomitant iris prolapse was present in most of the cases, we performed only suturing technique with 10/0 nylon. We did not perform keratoplasty in the follow-up periods because the corneal scars were not on the visual axis.

In terms of corrected final visual acuity, self-sealing corneal injuries had a better prognosis, whereas corneal perforations requiring surgical repair usually led to poor vision.^13^ Visual outcome following treatment of traumatic corneal perforation may not be optimal due to the presence of irregular astigmatism. Titiyal et al.^14^ reported that rigid gas-permeable contact lenses are better than spectacles in the visual rehabilitation of eyes that have scars caused by perforating corneal injuries. Unfortunately, we did not prescribe rigid contact lenses to our patients. Visual adaptation may occur with time in some ocular conditions such as trauma. Corneal remodeling and lessening of the astigmatism after suture removal might also have a positive effect on the visual acuity of patients who undergo corneal perforation repair in the chronic period. We waited for six months in order to maintain standardization and to allow time for visual adaptation and corneal remodeling after suture removal. Also, the ocular trauma score system is focused on the interpretation of visual acuity six months after trauma.^15^

Our study has several limitations. First of all, the sample size could have been larger. Second, early postoperative retinal and choroidal OCT measurements would have been beneficial. Lastly, visual acuity measurements should have been included after prescribing rigid contact lenses to the patients.

In conclusion, operated corneal perforations do not cause statistically significant retinal or choroidal alterations in the late postoperative period as measured by OCT. This statement does not mean that detailed retinal examination is not necessary after successfully operated corneal perforations. As expected, the low visual quality in the perforated eyes seems to be related to corneal problems. Further studies including corneal-scleral perforations should also provide insight into that related topic.

## Figures and Tables

**Table 1:**
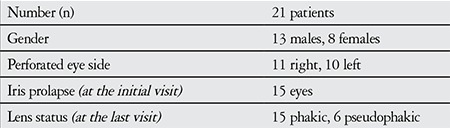
Demographic and clinical characteristics of the patients

**Table 2:**
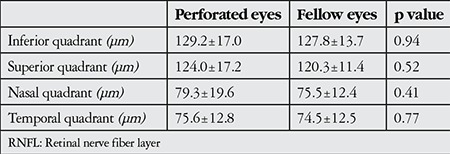
Quadrantal peripapillary retinal nerve fiber layer thickness values

**Table 3:**
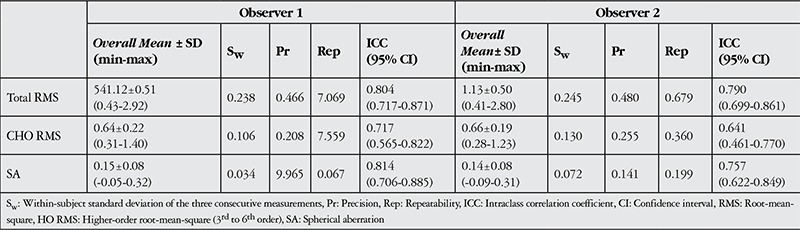
Peripapillary choroidal thickness and retinal vascular caliber measurements
